# Nasopharyngeal amyloidoma with osteolytic lesions leading to diagnosis of systemic light‐chain amyloidosis

**DOI:** 10.1002/ccr3.922

**Published:** 2017-04-13

**Authors:** Sandra Susanibar‐Adaniya, Pankaj Mathur, Rodolfo Henrich Lobo, Daisy Alapat, Manoj Kumar, Christoph Heuck, Sharmilan Thanendrarajan

**Affiliations:** ^1^ Department of HematologyMyeloma InstituteUniversity of Arkansas Medical SciencesLittle RockAR; ^2^ Department of PathologyUniversity of Arkansas Medical SciencesLittle RockAR; ^3^ Department of RadiologyUniversity of Arkansas Medical SciencesLittle RockAR

**Keywords:** Amyloidoma, nasopharyngeal tumor, osteolytic lesions, systemic light‐chain amyloidosis

## Abstract

Amyloidomas of the head and neck region are uncommon and generally considered a benign localized form of amyloidosis. Here, we describe “the unusual case of a young man” with a nasopharyngeal mass and osteolytic lesions caused by systemic light‐chain amyloidosis treated successfully with a combined surgical and chemotherapy approach.

A 44‐year‐old African‐American male presented to our emergency room after having experienced a syncopal episode and cervical pain after a jump from a 10′ wall. The patient reported nasal congestion, anosmia, several episodes of sinusitis in last six of months, and weight loss of more than 100 lb over the last 18 months. He further reported a longstanding history of cocaine, alcohol, and tobacco use. The physical examination was significant for a deviated septum and a visible mass inside the left nasal cavity resulting in a pronounced nasal voice with limited protrusion and lateral movement of the tongue. The laboratory work‐up was unremarkable, except for mildly elevated serum kappa free light chains (FLC) (13.9 mg/dL) and kappa/lambda ratio (11.0) and a urine immunofixation positive for kappa FLC.

A nasopharyngolaryngoscopy showed a large polypoid mass blocking most of the left nasal cavity causing nasal septum deviation and complete occlusion of the right nasal cavity. The CT showed a 9 cm large (sagittal axis), expansile, heterogeneous mass in the left nasal cavity with posterior extension to the nasopharynx and superior extension to the sphenoid sinus. The MRI confirmed a 7 × 5 cm mass in the nasal cavity extending into the left maxillary sinus and compressing the nasal septum to the right side (Fig. 1).

**Figure 1:**
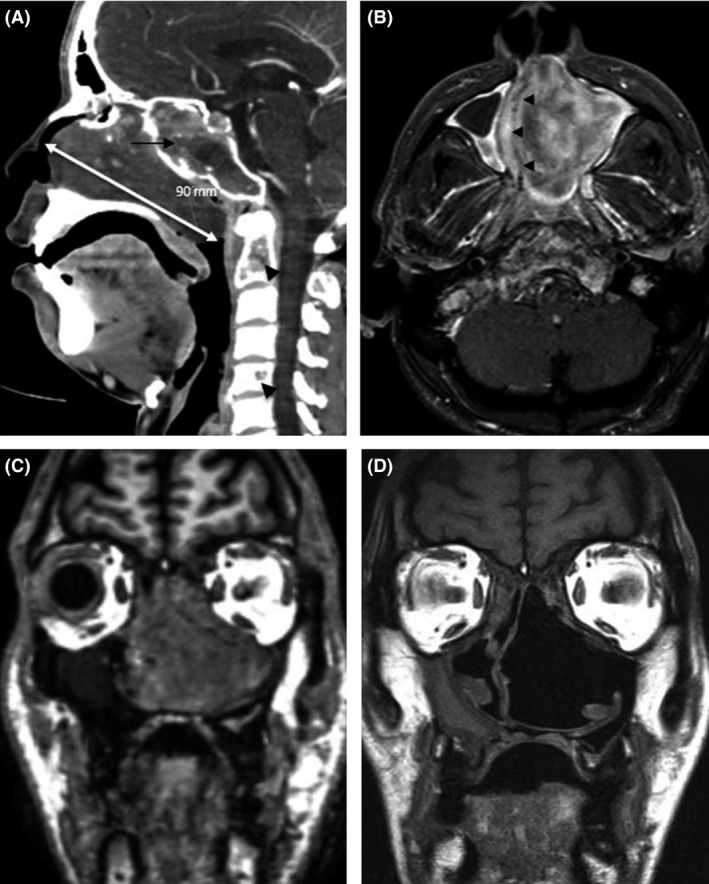
Images of nasopharyngeal mass (A) Preoperative CT. Sagittal view showed a 90 mm heterogeneous mass in the left nasal cavity (white double head arrow) with obstruction of the ethmoidal sinuses (black arrow) and osteolytic lesions in C2 and C5 (black arrowheads). (B) Preoperative postcontrast T1 image MRI study. Axial views showed marked nasal septum deviation (black arrowheads). (C and D) Pre‐ and postoperative MRI study after complete resection of the nasopharyngeal amyloidoma.

The patient had several lytic bone lesions involving the clivus, calvarium, and cervical, thoracic, and lumbar vertebral bodies on CT and MRI studies. The largest expansile bone lesion was at the C2 level measuring 3.0 × 1.1 cm completely replacing the dens and posterior elements of C2 vertebral body (Fig. 2). By FDG‐PET, both the mass in the nasal cavity and bone lesions showed no FDG avidity.

**Figure 2:**
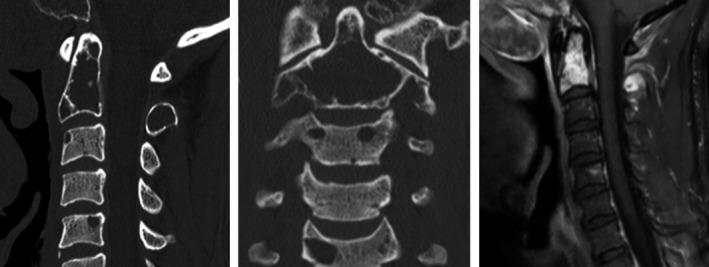
Images of C2 lesion MRI T1 images showed multiple bone lytic lesions. The largest at level of C2 involves the entire C2 dens and bilateral pedicles showing T1 hypointensity, and STIR hyperintensity.

The patient underwent neurosurgical intervention with an open C2 bone lesion biopsy with vertebral augmentation and endoscopic biopsy of the nasal mass. The biopsy of the C2 lesion revealed eosinophilic and amorphous, Congo red‐positive deposits and focal aggregates of CD138‐positive and kappa‐restricted plasma cells (Fig. 3). The biopsy of the nasopharyngeal mass also revealed eosinophilic and amorphous Congo red‐positive deposits (Fig. 4). Liquid chromatography‐mass spectrometric analysis of the deposits confirmed light‐chain (AL) kappa amyloid. The bone marrow aspirate and biopsy showed 4% and 5% of kappa‐restricted CD138‐positive plasma cells on aspirate and core biopsy, respectively. (Fig. 5). The phenotyping by flow cytometry showed 0.42% atypical plasma cells.

**Figure 3:**
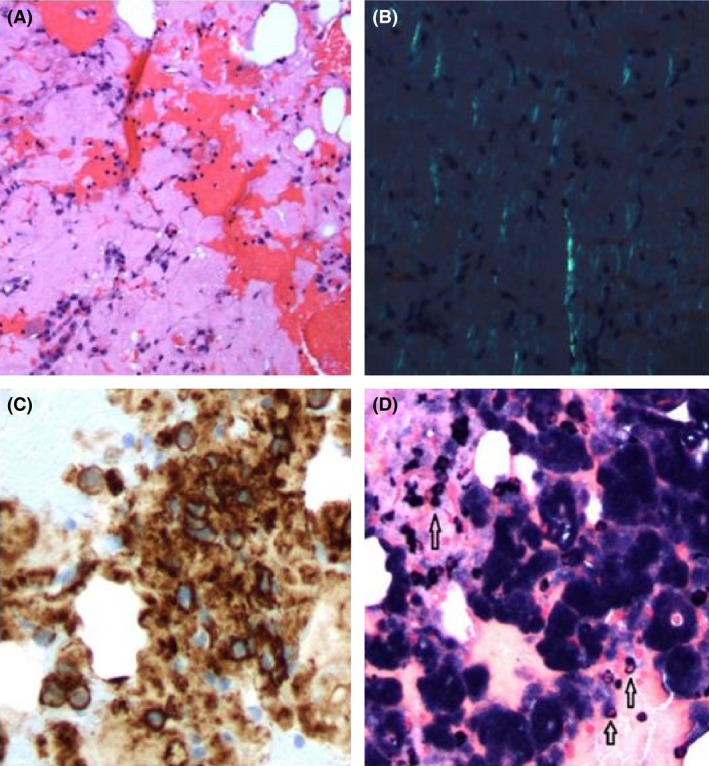
Biopsy of the C2 lesion (A) H&E stain revealed the deposition of a similar amorphous material as seen in the nasal mass biopsy. Scattered and small groups of plasma cells are identified interspersed among the deposits. (B) Congo red stain (20x) demonstrated apple‐green birefringence by polarized microscopy. (C) CD138 immunostain (20x) highlights the plasma cells present among the amyloid deposits. (D) In situ hybridization showed that the majority of plasma cells produce kappa light chain (white arrows).

**Figure 4:**
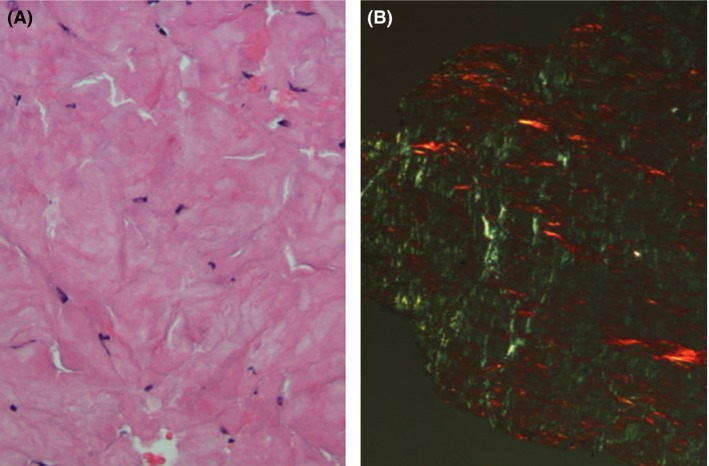
Biopsy of the nasopharyngeal mass (A) H&E stain (20x) showed the amorphous eosinophilic material with interspersed delicate vascular endothelial cells. (B) Congo red stain (20x) showed apple‐green birefringence of the amyloid deposits polarized light microscopy.

**Figure 5:**
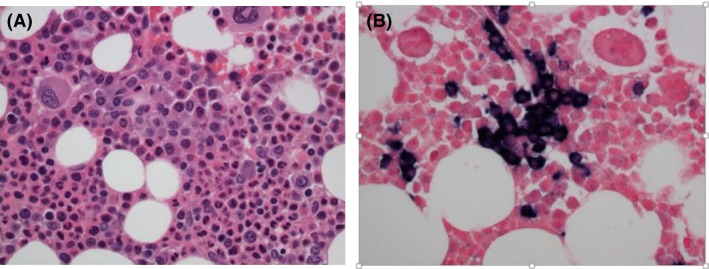
Bone marrow biopsy (A) H&E stain revealed a normocellular marrow with 5% plasma cells. (B) In situ hybridization showed that the plasma cells were kappa restricted.

These findings are consistent with a rare presentation of a large nasopharyngeal amyloidoma with extensive osteolytic lesions and low‐grade infiltration of myeloma cells in the bone marrow leading to diagnosis of primary, systemic light‐chain (AL) amyloidosis. The patient underwent extensive excision of the nasopharyngeal amyloidoma. Hereafter, he underwent induction chemotherapy with stem cell collection. In near future, he is planned for high‐dose chemotherapy and autologous stem cell transplantation.

Amyloidosis of the nasopharyngeal region is uncommon and represents <5% of the cases of amyloidosis of the head and neck region 1. Nasopharyngeal amyloidomas usually present at localized lesions and progression to systemic disease has not been reported previously. Bone involvement in the form of osteolytic lesions and vertebral compression fractures has been reported in primary systemic AL amyloidosis and is usually associated with visceral involvement. While the chronic use of inhalational cocaine by our patient opens the differential diagnosis to infectious, vasculitis, and other chronic inflammatory etiologies; the presence of diffuse osteolytic lesions guided our diagnostic work‐up into looking for plasma cell dyscrasias.

The main goal of this case was to create awareness that amyloidoma of head and neck region can present in rare cases as the initial manifestation of primary, systemic AL amyloidosis. Therefore, a comprehensive and interdisciplinary work‐up is necessary to look for other organ involvements and coexistence of plasma cell dyscrasia 2. Primary systemic (AL) amyloidosis is a fatal disease if not treated accordingly 2.

## Authorship

SSA, PM, CH, and ST: were involved in the clinical care of the patient. SSA and PM: did the literature review and manuscript preparation. RHL, DA, and MK: contributed to the acquisition and analysis of the data. CH and ST: critically reviewed the manuscript. All authors read and approved the final manuscript.

## Conflict of Interest

None declared.
